# Influenza virus infection affects insulin signaling, fatty acid-metabolizing enzyme expressions, and the tricarboxylic acid cycle in mice

**DOI:** 10.1038/s41598-020-67879-6

**Published:** 2020-07-02

**Authors:** Marumi Ohno, Toshiki Sekiya, Naoki Nomura, Taku ji Daito, Masashi Shingai, Hiroshi Kida

**Affiliations:** 1 Research Center for Zoonosis Control, Hokkaido University, Kita 20 Nishi 10, Kita-ku, Sapporo, 001-0020 Japan; 2 Global Station for Zoonosis Control, Global Institution for Collaborative Research and Education (GI-CoRE), Hokkaido University, Sapporo, Japan

**Keywords:** Influenza virus, Virology, Pathogenesis, Infection

## Abstract

Although the severity of influenza virus infections has been associated with host energy metabolism, the related mechanisms have not yet been clarified. Here we examined the effects of influenza virus infection on host energy metabolism in mice. After infecting mice with intranasal applications of 500 plaque-forming units of A/Puerto Rico/8/34 (H1N1; PR8) virus, the serum levels of most intermediates in the tricarboxylic acid (TCA) cycle and related metabolic pathways were significantly reduced. These data suggest that substrate supply to the TCA cycle is reduced under these conditions, rather than specific metabolic reactions being inhibited. Then, we focused on glucose and fatty acid metabolism that supply substrates to the TCA cycle. Akt phosphorylation following insulin injections was attenuated in the livers of PR8 virus-infected mice. Furthermore, glucose tolerance tests revealed that the PR8 virus-infected mice showed higher blood glucose levels than the vehicle-inoculated control mice. These results suggest that influenza virus infection impairs insulin signaling, which regulates glucose uptake. However, increases in the hepatic expressions of fatty acid-metabolizing enzymes suggest that fatty acids accumulate in liver cells of infected mice. Collectively, our data indicate that influenza virus infection dysregulates host energy metabolism. This line of investigation provides novel insights into the pathogenesis of influenza.

## Introduction

Influenza virus infection causes respiratory diseases and remains a major health concern, causing approximately half a million deaths per year globally^1^. Several types of antiviral drugs are commercially available for influenza treatment. However, influenza viruses change their protein structure constantly, thereby reducing their susceptibility to antiviral drugs, and prolonged treatment of patients receiving antivirals increases the chance of drug resistance of influenza viruses. In addition, the therapeutic effects of antiviral drugs are the highest when applied during the early stages of infection to prevent virus replication efficiently. Therefore, antiviral drug treatment is not suitable for patients with severe advanced stages of influenza. As long as we use antiviral drugs, these complications are always unavoidable. There is a need for the development of therapeutic strategies targeting host factors that are directly related to pathogenicity and symptoms.

Compared with influenza viruses themselves, host responses to virus infections are poorly understood. Although host immune responses, particularly excess cytokine secretion, are considered to be involved in influenza pathogenesis, the downstream responses of cytokine signaling have not yet been specified. In addition to inflammation, energy metabolism disorders, such as obesity, diabetes, and deficiencies of fatty acid oxidation, have been known to be related to influenza severity in mouse models and in humans^2–6^. Given the indispensable roles of inflammatory signaling in the development of a high-fat diet (HFD)-induced insulin resistance and imbalanced synthesis and oxidation of fatty acids^7,8^, it is hypothesized that influenza virus infection induces energy metabolism dysregulation in the host, following acute inflammation. To examine this hypothesis, we investigated metabolic changes by determining the serum levels of metabolites, insulin sensitivity in the liver, glucose availability, and hepatic gene expressions in the early stages of symptom onset as well as the lethal phase of influenza in a mouse model. The results of this study indicate that influenza virus infection dysregulates glucose and fatty acid metabolism and decreases tricarboxylic acid (TCA) cycle activity, leading to enhanced degradation of adenosine triphosphate (ATP) and guanosine triphosphate (GTP). We believe that this research provides novel insights into the pathogenesis of influenza and contributes to the development of novel influenza drugs.

## Results

### Metabolome analysis indicated reduced TCA cycle activity and enhanced purine degradation in mice with severe influenza

Upon infection of 500 plaque-forming units of PR8 virus, the mice showed significant body weight loss starting at 3 day post infection (1 dpi, 98.3% ± 2.1% in control mice, 99.5% ± 0.9% in PR8 virus-infected mice; 3 dpi, 100.2% ± 2.2% in control, 85.9% ± 0.7% in PR8 virus-infected mice; 6 dpi, 100.2% ± 2.4% in control mice, 75.5% ± 2.2% in PR8 virus-infected mice). Mice were sacrificed when weight loss reached a humane endpoint (25% at 6 dpi). To investigate the systemic effect of influenza virus infection on the host metabolic system, metabolome analyses were performed with serum samples collected at 1, 3, and 6 dpi for samples at very early-stage, onset of symptoms, and lethal phase during influenza, respectively. Metabolome analyses identified 74 metabolites in the serum samples of the control and PR8 virus-infected mice at all time points. Peak areas and relative ratios of all metabolites are provided in Supplemental Tables S1 and S2. Samples collected at 6 dpi showed the greatest effect of PR8 virus infection on serum metabolomes, as indicated by principle component analyses with high contribution rates of principle component 1 (PC1; 51.4%; Supplemental Fig. S1). Moreover, PC1 scores for the control and PR8 virus-infected mice were negative and positive, respectively, suggesting that the PC1 score was positively related to the effects of PR8 virus infection. Accordingly, metabolites with high positive and negative factor loading in PC1 tended to increase and decrease with PR8 virus infection, respectively (Table 1).

**Table 1:** Increased or decreased serum levels of metabolites in PR8 virus-infected mice at 6 dpi.

	Fold change (mean ± SEM)	Factor loading
**Increase**		
Inosine	2.68 ± 0.07	0.94
Xanthine	2.90 ± 0.48	0.81
Hypoxanthine	4.56 ± 1.19	0.73
**Decrease**		
Serotonin	0.78 ± 0.08	− 0.71
S-Adenosylhomocysteine	0.24 ± 0.05	− 0.72
Citric acid	0.71 ± 0.12	− 0.73
Cystine	0.48 ± 0.22	− 0.73
Asymmetric dimethylarginine	0.81 ± 0.08	− 0.76
Glutamic acid	0.81 ± 0.04	− 0.77
Adenosine	0.01 ± 0.00	− 0.80
Alanine	0.74 ± 0.06	− 0.80
Adenine	0.09 ± 0.00	− 0.80
Guanosine	0.25 ± 0.09	− 0.81
Glutathione	0.38 ± 0.13	− 0.83
Lactic acid	0.79 ± 0.06	− 0.83
Dimethylglycine	0.75 ± 0.06	− 0.83
Methionine	0.70 ± 0.04	− 0.84
Carnosine	0.59 ± 0.05	− 0.85
Tryptophan	0.80 ± 0.02	− 0.85
Aspartic acid	0.69 ± 0.05	− 0.86
Pyruvic acid	0.60 ± 0.09	− 0.87
Proline	0.62 ± 0.04	− 0.89
Succinic acid	0.26 ± 0.06	− 0.89
Argininosuccinic acid	0.35 ± 0.10	− 0.89
Adenylosuccinic acid	0.05 ± 0.01	− 0.89
Serine	0.52 ± 0.08	− 0.90
4-Aminobutyric acid	0.55 ± 0.10	− 0.90
2-Ketoglutaric acid	0.54 ± 0.07	− 0.91
Oxidized glutathione	0.24 ± 0.06	− 0.91
Carnitine	0.52 ± 0.07	− 0.92
Asparagine	0.49 ± 0.07	− 0.92
Tyrosine	0.58 ± 0.04	− 0.93
Glycine	0.50 ± 0.05	− 0.93
Malic acid	0.27 ± 0.09	− 0.94
Allantoin	0.59 ± 0.03	− 0.94
4-Hydroxyproline	0.24 ± 0.07	− 0.95
Orotic acid	0.46 ± 0.06	− 0.95
Guanosine monophosphate	0.01 ± 0.00	− 0.95
Citrulline	0.60 ± 0.04	− 0.96
Fumaric acid	0.24 ± 0.05	− 0.96
Cystathionine	0.36 ± 0.04	− 0.97

Metabolites with high factor loading (> 0.7) and significant differences (*p* < 0.05, *t*-test) were selected as significant metabolites and are listed here. The relative serum levels of each metabolite from PR8 virus-infected mice are presented as fold changes relative to those from control mice. Factor loading was defined for each metabolite as correlation coefficients between PC1 scores and metabolite levels that were normalized by autoscaling in each sample.

**Figure 1:**
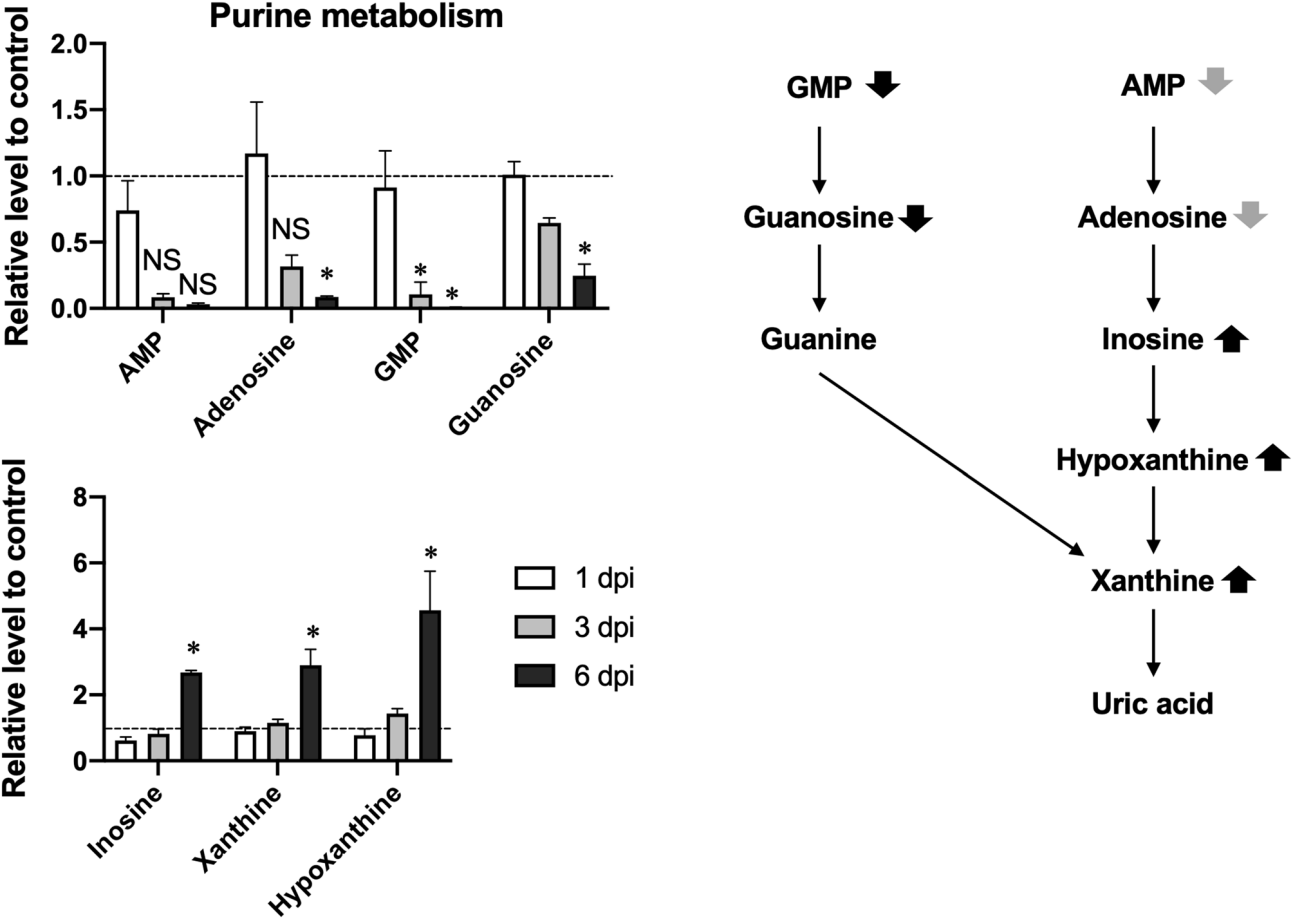
Relative serum levels of purine metabolites in the control vs. PR8 virus-infected mice at 1, 3, and 6 dpi. Mice were intranasally inoculated with PBS alone or PBS comprising PR8 virus, and serum samples were collected for metabolome analysis at 1, 3, and 6 dpi. The serum levels of purine metabolites in PR8 virus-infected mice were expressed relative to those in control mice at each time point. Bars represent mean ± SEM of 3 or 4 animals. In each panel, white, gray, and black bars indicate data from PR8 virus-infecetd mice at 1, 3, and 6 dpi, respectively; *p < 0.05, unpaired t-test using the Holm–Sidak correction method with an alpha value of 0.05, control vs. PR8 virus-infected mice at each time point. The right panel shows a schematic of the effects of PR8 virus infection on purine metabolism at 6 dpi. Upward and downward black arrows indicate significant increases and decreases, respectively. Downward gray arrows indicate observed but not significant decreases.

Relative increases in the serum levels of hypoxanthine, xanthine, and inosine were 4.56-, 2.90-, and 2.68-fold, respectively, in the PR8 virus-infected mice. These molecules are intermediates of purine degradation, and their increased serum levels are considered indicative of enhanced adenosine triphosphate (ATP) and guanosine triphosphate (GTP) catabolism. In agreement, the relative serum levels of the upstream purine metabolites adenosine monophosphate (AMP), adenosine, guanosine monophosphate (GMP), and guanosine, were decreased by 0.033-, 0.010-, 0.0075-, and 0.25-fold, respectively, at 6 dpi. Among them, the serum levels of AMP and adenosine did not differ significantly between the control and infected groups at any time point owing to large individual differences in the control groups (Fig. 1). Thus, the lethal phase of influenza is considered to be characterized by increased degradation of ATP and GTP.

**Figure 2:**
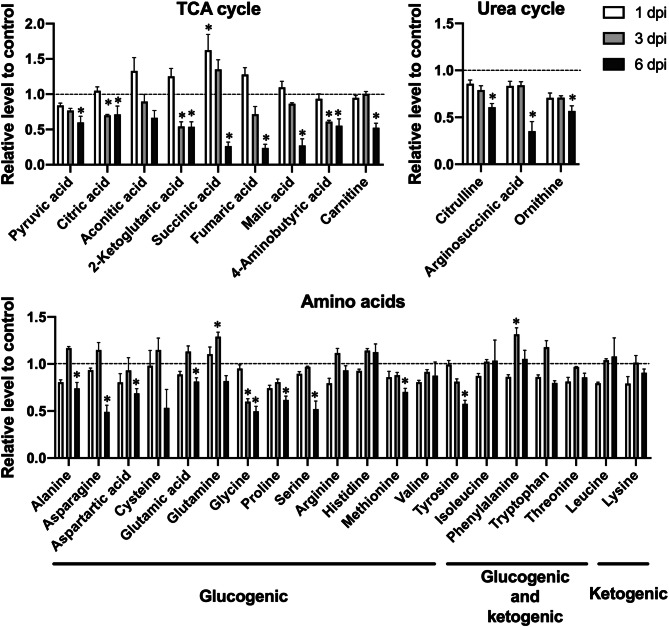
Relative serum levels of intermediates of the TCA cycle and related pathways in control vs. PR8 virus-infected mice at 1, 3, and 6 dpi. Mice were intranasally inoculated with PBS alone or PBS comprising PR8 virus, and serum samples were collected for metabolome analysis at 1, 3, and 6 dpi. The serum levels of TCA cycle metabolites in PR8 virus-infected mice are presented relative to those in control mice at each time point. Each panels show metabolites of the TCA cycle, urea cycle, and amino acid metabolism, respectively. Bars represent mean ± SEM of 3 or 4 animals. In each panel, white, gray, and black bars indicate data from PR8 virus-infected mice at 1, 3, and 6 dpi, respectively; *p < 0.05, unpaired t test using the Holm–Sidak correction method with an alpha value of 0.05, control vs. PR8 virus-infected mice at each time point.

**Figure 3:**
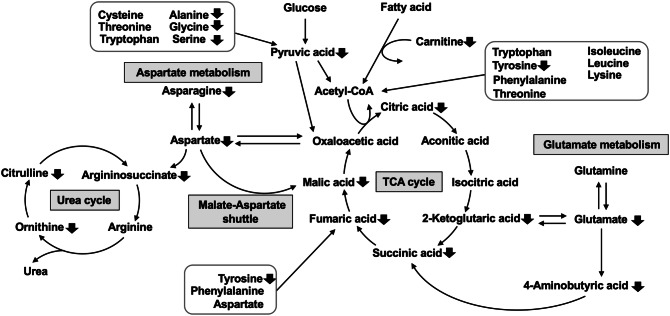
Serum metabolome analysis. Schematic of the effects of PR8 virus infection on the TCA cycle and related metabolic pathways at 6 dpi; upward and downward arrows indicate significant increases and decreases, respectively.

Metabolites that were present at reduced levels in the sera of PR8 virus-infected mice were mainly related to the TCA cycle, urea cycle, and amino acid metabolism, as indicated by the serum levels of metabolite in these pathways at 1, 3, and 6 dpi (Fig. 2). The effects of 6-day PR8 virus infections on the serum levels of metabolite are summarized in Fig. 3. The levels of TCA cycle metabolites pyruvic acid, 2-ketoglutaric acid, fumaric acid, and malic acid were significantly reduced in PR8 virus-infected mice at 6 dpi (0.60-, 0.54-, 0.24-, and 0.27-fold, respectively). Succinic acid levels were significantly increased by 1.62-fold at 1 dpi but decreased by 0.26-fold at 6 dpi in PR8 virus-infected mice. In addition, 4-aminobutyric acid, which is synthesized from glutamic acid and converted to succinic acid, was present at reduced in PR8 virus-infected mice at 3 and 6 dpi (0.61- and 0.55-fold, respectively). The levels of urea cycle metabolites argininosuccinic acid, ornithine, and citrulline were decreased by 0.35-, 0.57-, and 0.61-fold, respectively, in PR8 virus-infected mice at 6 dpi, whereas the level of arginine was not altered significantly at any time point. Given that the levels of most metabolites were significantly reduced in the TCA cycle and related pathways, we suggest that PR8 virus infection reduces flux through these pathways, particularly through the TCA cycle.

As for amino acids, while the serum levels of ketogenic amino acids did not show any apparent decreases, those of glucogenic amino acids were significantly reduced in PR8 virus-infected mice at 6 dpi. Specifically, the serum levels of asparagine, glycine, proline, serine, and tyrosine were significantly decreased by 0.49-, 0.50-, 0.62-, 0.52-, and 0.58-fold, respectively. Furthermore, to estimate the effect of PR8 virus infection on glutaminolysis in which glutamine is converted to 2-ketoglutaric acid via glutamic acid and enters the TCA cycle, we compared the ratio of the peak areas of glutamic acid to those of glutamine and the ratio of the peak areas of ketoglutaric acid to those of glutamic acid. While the ratio of glutamic acid to glutamine was not changed by the infection, the ratio of 2-ketoglutaric acid to glutamic acid was significantly decreased by 0.48- and 0.65-fold, respectively, at 3 and 6 dpi (Supplemental Table S3). Therefore, it was indicated that glutaminolysis contribution to the TCA cycle was attenuated by influenza virus infection.

Pathway analysis using MetaboAnalyst further confirmed that the TCA cycle and related pathways were significantly affected by PR8 virus infection at 6 dpi. The top 10 pathways are listed in Table 2. Given that the TCA cycle generates GTP and contributes electrons to mitochondrial oxidative phosphorylation to generate a substantial amount of ATP, suppression of the TCA cycle in PR8 virus-infected mice is considered to cause imbalanced synthesis and degradation of ATP and GTP. Moreover, these data suggest that suppression of the TCA cycle was due to reduced substrate supply to the pathway due to PR8 virus infection, rather than inhibition of specific metabolic reactions. TCA cycle flux is associated with glucose and fatty acid metabolism because both these energy sources are eventually converted to acetyl-CoA, which enters the TCA cycle. Because the liver plays a predominant role in whole-body energy metabolism, we further investigated the effect of PR8 virus infection on liver function in terms of glucose and fatty acid metabolism.

**Table 2:** Top 10 pathways.

	Total	Hits	Raw p	− log(p)	Holm p
Citrate cycle (TCA cycle)	20	6	8.75E−05	9.3443	0.003236
Propanoate metabolism	23	1	0.00015921	8.7453	0.0057315
Glutathione metabolism	28	6	0.00020376	8.4985	0.0071317
Alanine, aspartate, and glutamate metabolism	28	13	0.00024666	8.3075	0.0083866
Purine metabolism	66	12	0.00056079	7.4862	0.018506
Tyrosine metabolism	42	3	0.00056331	7.4817	0.018506
Glyoxylate and dicarboxylate metabolism	32	7	0.00093261	6.9775	0.028911
Arginine biosynthesis	14	9	0.00098009	6.9279	0.029403
Butanoate metabolism	15	4	0.0011932	6.7312	0.034602
Porphyrin and chlorophyll metabolism	30	2	0.0014728	6.5206	0.041238

Pathway analyses were performed using MetaboAnalyst software to identify pathways that were significantly affected by PR8 virus infection at 6 dpi. In the table, Total is total number of compounds in the pathways; Hit is number of compound matched from our data; Raw *p* is the original *p* value calculated from pathway analysis; Holm p is *p* values adjusted by the Holm–Bonferroni method.

### Influenza virus infection impairs insulin sensitivity in the liver

**Figure 4:**
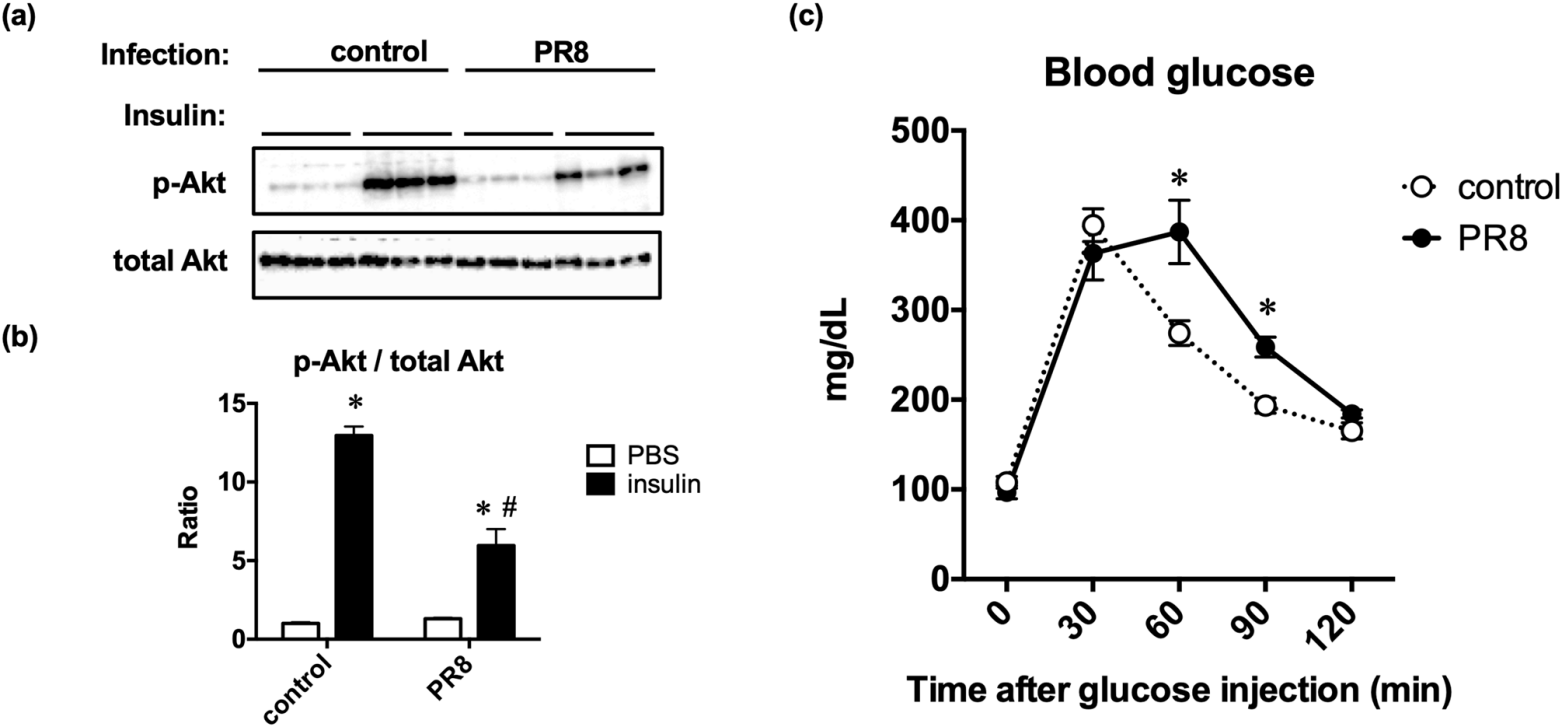
Effects of PR8 virus infection on insulin-induced Akt phosphorylation. Mice were intranasally inoculated with PBS alone or PBS comprising PR8 virus. At 6 dpi, the mice were intraperitoneally injected with PBS or insulin after overnight fasting, and liver samples were collected after 15 min for whole lysate preparation. Western blotting was performed to quantitate phosphorylated and total Akt protein levels in lysates. (**a**) A representative Western blotting analysis shows total Akt and Akt phosphorylated at Ser473 on the same membrane that was sequentially immunoblotted with corresponding antibodies. (**b**) Relative Akt phosphorylation levels were calculated from band densities and expressed relative to data from PBS-treated control mice. Bars represent mean ± SEM of 3 animals. White and black bars indicate data from PBS- and insulin-treated mice, respectively; *p* < 0.05, two-way ANOVA, PBS vs*.* insulin (*), control vs. PR8 virus-infected mice (#). (**c**) Mice were intranasally inoculated with PBS alone or PBS comprising PR8 virus. At 6 dpi, GTT was performed after overnight fasting. To this end, mice were intraperitoneally injected with glucose, and their blood glucose levels were sequentially measured at 0, 30, 60, and 90 min post injection. Dots represents mean ± SEM of 5 animals; *p < 0.05, unpaired t test using the Holm–Sidak correction method with an alpha value of 0.05, control vs. PR8 virus-infected mice at each time point.

Insulin regulates cellular glucose uptake from the blood and intracellular glycolysis^9,10^, which supplies substrates to the TCA cycle. Upon insulin binding to the insulin receptor on the cell surface, the signal is transduced to downstream molecules and phosphorylates Akt. Phosphorylated Akt activates glucose transporters to increase glucose uptake. Therefore, phosphorylation of Akt is a good indicator of activation of insulin signaling. Here we examined the effect of influenza virus infection on insulin sensitivity in the livers according to ratios of insulin-induced phosphorylation of Akt. Western blotting analyses (Fig. 4a) of phosphorylated and total Akt in whole liver lysates of control and PR8 virus-infected mice at 6 dpi revealed that the ratios of phosphorylated Akt to total Akt were increased by 13.0-fold after insulin treatments in control mice, but this ratio was increased by only 4.6-fold in PR8 virus-infected mice (Fig. 4b). Similar experiments were performed with the livers collected at other time points. At 3 dpi, Akt phosphorylation was clearly inhibited by PR8 virus infection, but no clear differences were observed in samples collected at 1 dpi (Supplemental Fig. S2). Taken together, these results demonstrated that influenza virus infection impairs insulin actions in the liver.

We also performed glucose tolerance tests (GTT) in control and PR8 virus-infected mice at 6 dpi to investigate whether PR8 virus infection affected glucose uptake in response to high doses of glucose (Fig. 4c). We found no significant differences in fasting blood glucose levels between the groups. Moreover, at 30 min after glucose injections, the control and PR8 virus-infected mice showed similar blood glucose levels (394.4 ± 18.2 mg/dL vs. 363.2 ± 29.7 mg/dL). Subsequently, blood glucose levels were rapidly decreased in control mice (60 min, 274.2 ± 13.7 mg/dL; 90 min, 193.4 ± 8.5 mg/dL; 120 min, 165.4 ± 9.0 mg/dL). Conversely, decreases in blood glucose levels were delayed and higher blood glucose levels were observed in PR8 virus-infected mice, compared with the control mice, at all time points (60 min, 387.0 ± 35.4 mg/dL; 90 min, 258.6 ± 11.1 mg/dL; 120 min, 184.2 ± 4.4 mg/dL). Significant differences in blood glucose levels were identified at 60 and 90 min between the groups (*p* < 0.05, two-way ANOVA). These results indicate that PR8 virus infection impairs insulin signaling in the liver and induces a tendency toward glucose intolerance, potentially reflecting reduced glucose uptake.

### Fatty acid accumulation in the liver of infected mice was suggested by gene expression assays

**Figure 5:**
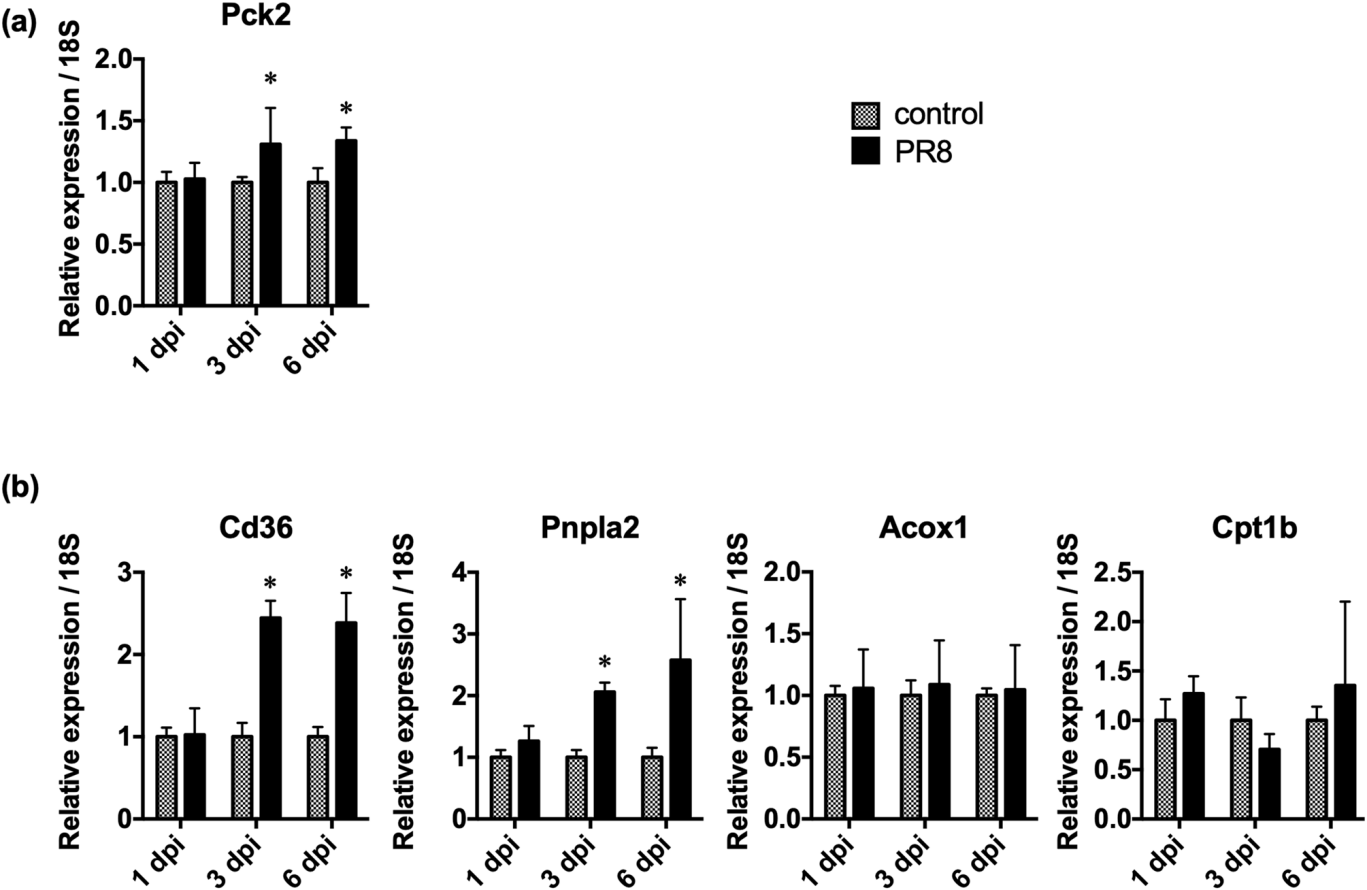
Expressions of energy metabolism-related genes in the liver. Mice were intranasally inoculated with PBS alone or PBS comprising PR8 virus, and liver samples were collected at 1, 3, and 6 dpi. The gene expressions of *Pck2*, *Cd36*, *Pnpla2*, *Acox1*, and *Cpt1b* were normalized with that of 18S from real-time PCR analyses. Gene expressions of the PR8 virus-infected mice are presented as fold changes relative to those of the control mice at each time point. Bars represent means ± SEM of 3 or 4 animals. Gray and black bars indicate data from control and PR8 virus-infected mice, respectively; *p < 0.05, unpaired t test using the Holm–Sidak correction method with an alpha value of 0.05, control vs. PR8 virus-infected mice at each time point.

We also investigated the effects of PR8 virus infection on the hepatic expressions of genes that are transcriptionally regulated by insulin signaling (Fig. 5). *Pck2*, which encodes a gluconeogenic enzyme, was expressed at slightly but significantly increased (1.3-fold) levels in the liver of infected mice at 3 and 6 dpi (Fig. 5a). This gene is negatively regulated by insulin signaling through inactivation of a transcriptional factor forkhead box-containing protein O sub-family 1 (FoxO1) which reduces glycolysis and activates gluconeogenesis^11^. Therefore, the increase in *Pck2* expression in the present study also suggests reduced insulin activity or sensitivity in PR8 virus-infected mice.

In addition to glucose, fatty acids are important sources of acetyl-CoA for the TCA cycle. Fatty acid metabolism was previously reported to be inhibited by influenza virus infection^12,13^. Therefore, we investigated whether PR8 virus infection altered the hepatic expressions of genes encoding fatty acid-metabolizing enzymes (Fig. 5b). *Cd36* and *Pnpla2* play important roles in fatty acid transport and lipolysis, respectively, and their gene expressions are downregulated by insulin signaling^14,15^. Here, *Cd36* was expressed at significantly increased levels in the livers of PR8 virus-infected mice, with 2.44- and 2.38-fold increases at 3 and 6 dpi, respectively. In addition, *Pnpla* was significantly induced by 2.06- and 2.57-fold at 3 and 6 dpi, respectively. Significant increases in the expressions of *Cd36* and *Pnpla2* as well as of *Pck2* suggested the attenuation of insulin signaling in the liver of PR8 virus-infected mice. Conversely, we observed no changes in the expressions of *Acox1* and *Cpt1β*, which encode key enzymes of fatty acid β-oxidation. In addition, the serum levels of carnitine were significantly decreased by 0.52-fold at 6 dpi (Fig. 2). Because carnitine is involved in fatty acid transport into mitochondria, the observed decrease would possibly reduce fatty acid β-oxidation in mitochondria. These changes suggest fatty acid accumulation in the liver cells of PR8 virus-infected mice, although further analyses on protein expressions and enzymatic activities are warranted in the future.

## Discussion

In the present study, metabolome analyses demonstrated that PR8 virus infection decreases the serum levels of most TCA cycle intermediates and metabolites in the related metabolic pathways, suggesting reduced flux through the TCA cycle. Because the TCA cycle greatly contributes electrons for mitochondrial oxidative phosphorylation, suppression of the TCA cycle leads to reduced ATP synthesis. Hence, the rates of ATP and GTP degradation were possibly greater than the respective rates of their synthesis in PR8 virus-infected mice, leading to significant increases in the serum levels of metabolites, such as xanthine, hypoxanthine, and inosine. Energy depletion indicated by the reduced levels of ATP and GTP has been described in several studies on infectious diseases and endotoxemia^16–18^. Reduced ATP levels in blood and various organs, including the liver, have been previously associated with influenza virus infection in a mouse model^16^. Decreased GTP levels have been shown in the lung tissues of rabbits treated with bacterial endotoxin lipopolysaccharide^17^. Increases in the serum levels of hypoxanthine and inosine have also been reported in patients with primary dengue virus infection at the febrile stage, suggesting an imbalance of ATP and/or GTP synthesis and degradation during the acute stage of the dengue fever^18^. These studies and ours indicate that regulatory energy depletion is a common symptom of infectious diseases.

TCA cycle flux is associated with glucose and fatty acid metabolism because these energy sources are eventually converted to acetyl-CoA for entry into the TCA cycle. If either pathway is inhibited, the other pathway becomes activated to compensate for the reduced substrate supply to the TCA cycle. In the case of HFD-induced obese mice with insulin resistance, fatty acid oxidation is increased in a leptin-dependent manner, and TCA cycle activity is enhanced^19,20^. Conversely, *db*/*db* mice, a well-known mouse model of type 2 diabetes, reportedly developed insulin resistance without activation of fatty acid oxidation due to lack of leptin receptor and showed reduced serum levels of the TCA cycle intermediates citric acid, 2-ketoglutaric acid, malic acid, and fumaric acid with disease progression^21^. In the present study, no changes in the expression of *Cpt1* or *Acox1* in the liver suggest that fatty acid oxidation was not enhanced in PR8 virus-infected mice despite insulin resistance. Although the effect of PR8 virus infection on fatty acid oxidation was not evaluated in the present study, reduced fatty acid oxidation during influenza has been demonstrated previously^12,13^. If both insulin signaling and fatty acid oxidation are inhibited during influenza, it might result in suppression of the TCA cycle due to reduced substrate supply. Given significant body weight loss in PR8 virus-infected mice, however, decrease in food intake during influenza could affect the serum levels of the TCA cycle and related pathways. Further investigations, such as studies with pair-fed control and lower titer of virus, will provide helpful information to confirm the underlying association.

Here we demonstrated for the first time that influenza virus infection impairs insulin signaling in liver tissues, which critically regulates glucose metabolism^9,10^, and induces a tendency toward glucose intolerance. Importantly, Nagao et al., reported a high blood glucose level (over 150 mg/dL) as one of the worst prognostic factors in pediatric patients with influenza-associated encephalopathy^22^. In addition to the elevated glucocorticoid levels, as speculated by Nagao et al., our study suggests the impairment of insulin signaling as a mechanism of elevated blood glucose levels in children with severe influenza. Conversely, another study reported that glucose uptake was activated in the lungs of patients with respiratory viral infections, including influenza virus infection^23^. This discrepancy may be associated with tissue-specific metabolic changes in response to influenza virus infection. However, given the direct activating effect of influenza virus proliferation on glucose metabolism in cultured Madin–Darby Canine Kidney cells^24^, the hepatic insulin resistance demonstrated here could have been induced by host factors and not the virus itself. Upon intranasal infection of PR8 virus, proliferation of the virus occurs mainly in the lung and not in the liver of mice^25^. Hence, the hepatic insulin resistance observed herein is possibly induced by host factors, such as systemically secreted cytokines. The direct roles of cytokines on insulin signaling have been demonstrated in a study showing that the proinflammatory cytokine interleukin-6 (IL-6) inhibited insulin-dependent phosphorylation of Akt in HepG2 cells and human primary hepatocytes^26^. Furthermore, IL-6 treatments reportedly impaired insulin signaling and inhibited insulin-induced decreases in blood glucose levels in mice^27^; in this previous experiment, the serum levels of IL-6 reached approximately 125 pg/mL, which was comparable to that observed herein in PR8 virus-infected mice at 3 and 6 dpi when reduced insulin-induced phosphorylation of Akt was observed (Supplemental Fig. S3, 109.3 and 137.1 pg/mL at 3 and 6 dpi, respectively). Thus, we suggest that this cytokine is one of host factor candidates that affect insulin resistance, particularly in the liver. In addition to IL-6, interferon-gamma (IFN-γ) was reportedly induced by murine cytomegalovirus infections and was recently shown to induce insulin resistance in skeletal muscle^28^. Besides IL-6, the serum levels of IFN-γ were elevated in PR8 virus-infected mice at 6 dpi but were very low at 3 dpi in our study (Supplemental Fig. S3; 1.391 and 313.4 pg/mL at 3 and 6 dpi, respectively). Therefore, we propose that influenza virus infection induces hepatic insulin resistance through systemic cytokine secretion.

As stated above, in addition to glucose, fatty acids are important sources of the TCA cycle substrate acetyl-CoA. Reduced fatty acid metabolism during influenza has been reported in previous reports showing mitochondrial abnormalities, decreases in the expressions of relevant enzymes, and hepatic steatosis in mice^12,13^. Consistently, our gene expression data indicate altered fatty acid metabolism in the livers of PR8 virus-infected mice. In particular, the transcription levels of the fatty acid transporter *Cd36* and the lipolytic enzyme *Pnpla2* were upregulated at 3 and 6 dpi, possibly elevating extracellular fatty acid delivery and stored triglyceride lysis in liver cells. However, no changes were observed in the mRNA expressions of *Acox1* and *Cpt1b*. These encoded enzymes are rate limiting factors for fatty acid β-oxidation in peroxisomes and mitochondria, respectively. Taken together, our data suggest that fatty acids are accumulated in the cytoplasm of liver cells in influenza virus-infected mice. Although anorexia due to influenza virus infection may induce similar alterations in gene expressions and subsequent hepatic steatosis, the findings of pair-fed studies on influenza eliminated the possibility that starvation is solely responsible for these changes^12,13^. Moreover, fasting generally increases the mRNA expressions of *Acox1* and *Cpt1b*, thereby discriminating the effects of influenza virus infection on fatty acid metabolism from those of starvation^29,30^. Fatty acid accumulation can induce mitochondrial dysfunction, as reported previously in cultured hepatocytes^31^. Mitochondrial dysfunction increases oxidative stress via reactive oxygen species production. Reactive oxygen species and diacylglycerol, converted from triacylglycerol by adipose triglyceride lipase (encoded by *Pnpla2*), inhibit insulin signaling^32^. It is suggested that accumulation of intracellular fatty acids triggers and complicates insulin resistance in the liver of infected mice.

Given the higher influenza-associated mortality and morbidity rates in patients and mice with energy metabolism disorders^2–6^, energy metabolism dysregulation induced by influenza virus infection is possibly associated with pathogenicity. Increased mortality rates with influenza have been reported in diet-induced obese mice which probably had insulin resistance as well as in long-chain acyl-CoA dehydrogenase-knockout mice, which had reduced mitochondrial fatty acid oxidation^2,5^. Interestingly, both these mouse models presented with subdued immune responses to virus infection and similar or lower lung virus titers compared with the control mice. Hence, increased influenza severity in animals and patients with energy metabolism disorders can be explained neither by the increased levels of inflammatory cytokines nor by the reduced clearance of pathogens^2,5^. As described above, dysregulation of energy metabolism appears to be a downstream response to cytokine and inflammatory signaling and thus could be more directly associated with the pathogenesis and severity of influenza. Previous cohort studies have demonstrated that antiinflammatory corticosteroids have no beneficial effects and that these corticosteroids can exacerbate outcomes in patients with severe influenza^33^. Given that glucocorticoids inhibit insulin signaling as well as cytokine-dependent pathogen clearance^34,35^, corticosteroid treatments could confound pathogenicity. Thus, improvements in host energy metabolism would be a novel therapeutic target for severe influenza rather than immune suppression. Moreover, this concept could be expanded to other infectious diseases because energy metabolism dysregulation is possibly induced by host factors, such as proinflammatory cytokines, and not by the viruses themselves. Given the recent emerging infectious diseases such as the pandemic influenza in 2009 and severe pneumonia by the novel corona virus in 2019, the importance of developing novel therapeutic drugs that target host factors against common symptoms of various diseases is important.

Infants and children younger than 5 years of age are generally at high risks of mortality and morbidity due to influenza compared with middle-aged adults^36^. Previously, a negative correlation of nasal lavage and plasma inflammatory cytokine levels with age has been reported and thought to be a reason for the high mortality and severe illness caused by influenza virus infection in young children^37,38^. In addition to cytokine responses, age-specific features of energy metabolism could provide insights into the pathogenesis of influenza at different ages. In pediatric patients with type I diabetes, the youngest population (0–5 years of age) reportedly showed the highest prevalence of diabetic ketoacidosis, which has an impact on morbidity and mortality^39^, indicating that attenuation of insulin signaling by virus infection could result in more severe symptoms in such populations.

The present study provides novel insights into host responses during influenza. Based on the findings of the present and previous studies, we conclude that influenza virus infection induces dysregulation of both insulin-regulating glucose metabolism and fatty acid oxidation, leading to decreased TCA activity. In future studies, we aim to investigate the effect of influenza virus infection at lethal and sublethal doses of various virus strains for further understanding of influenza pathogenesis and examine the therapeutic effects of interventions that improve host energy metabolism.

## Materials and methods

### Materials

Phosphate-buffered saline (PBS) was purchased from Gibco/Life technologies (Carlsbad, CA). Tris-buffered saline (TBS) tablets were purchased from Takara Bio (Otsu, Japan). Urea, Tween 20, and glucose were purchased from Sigma-Aldrich (St Louis, MO). Sodium dodecyl sulfate (SDS) was purchased from WAKO (Tokyo, Japan).

### Virus

Influenza virus A/Puerto Rico/8/34 (H1N1; PR8) was kindly provided by the National Institute of Infectious Disease in Japan. The virus was propagated in 10-day-old embryonated chicken eggs at 35 °C for 48 h, and aliquots of collected allantoic fluids were stored at − 80 °C until further analysis.

### Mouse

Male C57BL/6 mice were purchased from Hokudo (Sapporo, Japan) and were kept at a BSL-2 laboratory at the Research Center for Zoonosis Control, Hokkaido University, under standard laboratory conditions (room temperature 22 °C ± 2 °C, relative humidity 50% ± 10%) and a 12/12-h light/dark cycle. The mice were administered a standard CE-2 chow diet purchased from CLEA Japan (Sapporo, Japan) with water ad libtum. Experiments were performed on 9–14 week-old mice.

### Virus infection and sample collection

PR8 virus particles at 500 plaque-forming units in 50 µL of PBS or PBS only (control) were intranasally inoculated into the mice under inhalation anesthesia with isoflurane. At 1, 3, or 6 dpi, the mice were euthanized, and their liver and blood samples were collected. Blood samples were incubated at room temperature for 1 h to clot and were then centrifuged at 1,000*g* for 20 min. Supernatants were collected as serum and were stored at − 20 °C until further analysis. Tissue samples were stored at − 80 °C until further analysis. To evaluate insulin sensitivity, the mice were administered insulin (Humulin R, Eli Lilly, Indianapolis, IN) at 2 U/kg body weight intraperitoneally at 6 dpi after overnight fasting. After 15 min, the mice were euthanized, and their liver samples were collected and frozen immediately in liquid nitrogen. Liver samples were stored at − 80 °C until further analysis.

### Metabolome analysis

Targeted global metabolome analyses were performed by Shimadzu Techno Research (Kyoto, Japan) using liquid chromatography–tandem mass spectrometry (LC–MS/MS). Briefly, after the addition of an internal standard (2-isopropylmalic acid) to the serum samples, sample preparation was conducted using a series of hydrochloride and acetonitrile extractions. After centrifugation at 10,000 rpm for 5 min at room temperature, the supernatants were divided into two tubes: one was used for analyses of AMP, GMP, aconitic acid, citric acid, and fumaric acid, whereas in the other one, the supernatant was mixed with hydrochloride and used for analysis of other compounds. Chromatographic separations were performed on a 2 Discovery HS F5-3 column (2.1 mm × 150 mm, 3 μm, Sigma-Aldrich). The oven temperature for the column was maintained at 40 °C. Mobile phases A and B were 0.1% formic acid–water solution and 0.1% formic acid–acetonitrile, respectively. Separation was performed using gradient elution at a flow rate of 0.25 mL/min with a Nexera UHPLC system (Shimadzu). Compounds were eluted by changing proportions of mobile phase B as follows: 0% (0–2 min), 0%–25% B (2–5 min), 25%–35% B (5–11 min), 35%–95% (11–15 min), 95% (15–20 min), 95%–0% (20–20.1 min), and 0% (20.1–25 min). Compounds were detected using an LCMS-8050 (Shimadzu) instrument with electrospray ionization. The peak areas of each compound were measured using Labsolutions (Shimadzu) and were normalized to that of an internal standard. The metabolome analyses identified 74 molecules in our sample sets. Among them, cysteine, cytosine, and methionine sulfoxide were not detected in some samples from PR8 virus-infected mice at 3 and 6 dpi (Supplemental Table S1). To examine the effects of PR8 virus infection on the serum levels of various compounds, the relative peak areas for PR8 virus-infected mice were expressed as fold changes relative to the average peak areas for the control mice at corresponding time points. The peak areas and relative ratios are provided in Supplemental Table S1 and S2.

### Serum metabolome data analysis using MetaboAnalyst

Utilizing peak areas of 74 molecules, principal component analysis and pathway analysis were performed with MetaboAnalyst (https://www.metaboanalyst.ca). The values were normalized by autoscaling function. Missing values in cysteine, cytosine, and methionine sulfoxide were replaced by small values (a half of the minimum positive values in the original data). Factor loading was defined for each metabolite as the correlation coefficient between the PC1 score and the level of the metabolite after normalizing by autoscaling for each sample. Metabolites with high factor loading (> 0.7) and significant differences (*p* < 0.05, *t*-test) were selected as significant metabolites and are listed in Table 1. Pathway analyses were performed based on the Kyoto Encyclopedia of Genes and Genomes (https://www.genome.jp/kegg/), and the most significantly altered metabolic pathways in PR8 virus-infected mice were identified. The top 10 pathways identified are listed in Table 2.

### Evaluation of phosphorylated Akt by Western blotting

Liver samples were homogenized in TBS comprising 8 M urea and 1% SDS using an ultrasonic homogenizer (Q125 sonicator, Qsonica, Newtown, CT) and centrifuged at 15,000*g* for 10 min at 10 °C to obtain supernatants as whole liver lysates. Protein levels were measured using Pierce BCA protein assay kits (Thermo Fisher Scientific, Waltham, MA). Samples for Western blotting were prepared by mixing whole liver lysates with the NuPAGE LDS Sample buffer (Thermo Fisher Scientific) and heating at 70 °C for 10 min. Subsequently, 10 μg aliquots of total protein were separated using 10% SDS–polyacrylamide gel electrophoresis (SDS–PAGE) and were transferred to polyvinylidene difluoride membranes. After blocking with 5% nonfat dry milk in TBS buffer comprising 0.1% Tween 20 (TBST), the membranes were probed with an anti-Akt phosphorylated at Ser473 antibody (1:1,000, #9271, Cell Signaling Technology, Beverly, MA) or an anti-Akt antibody (1:1,000, Cell Signaling Technology) in TBST buffer comprising 5% bovine serum albumin overnight at 4 °C. The membranes were then treated with secondary horseradish peroxidase-conjugated antirabbit antibody (1:5,000, sc-2357, Santa Cruz) for 1 h at room temperature. Protein bands on membranes were detected using SuperSignal West Femto Maximum Sensitivity Substrate (Thermo Fisher Scientific) and an ImageQuant LAS4000 system (GE, Buckinghamshire, UK). The bands were quantified using the ImageQuant TL analysis system (GE). Full-length images are provided in Supplemental Fig. S4.

### GTT

The control and PR8 virus-infected mice received intraperitoneal injections of 20% glucose solution in PBS at 2 g/kg body weight after overnight fasting. Blood was collected from tail veins at 0, 30, 60, and 90 min after injections, and plasma glucose concentrations were measured using an Accu-chek glucose meter (Roche Diagnostic, Mannheim, Germany).

### Measurement of selected gene expressions using real-time PCR

Total RNA was extracted from tissue samples using TRIzol (Thermo Fisher Scientific) and was used for cDNA synthesis using High-Capacity cDNA Reverse Transcription Kits (Thermo Fisher Scientific), according to the manufacturer’s instructions. The gene expressions of *Cluster of differentiation 36* (*Cd36*, Mm00432403_m1), *Patatin Like Phospholipase Domain Containing 2* (*Pnpla2*, Mm00503040_m1), *Acyl-CoA Oxidase 1* (*Acox1*, Mm01246834_m1), *Carnitine Palmitoyltransferase 1 Beta* (*Cpt1b*, Mm00487191_g1), and *Phosphoenolpyruvate Carboxykinase* 2 (*Pck2*, Mm00551411_m1) were quantified using real-time PCR with a StepOne Real-Time PCR system (Applied Biosystems, Foster City, CA) with TaqMan probes (Applied Biosystems). The obtained gene expressions were normalized to those of *18S* (Mm03928990_g1) from the same samples, and relative expressions were calculated using the comparative Ct method (ΔΔCt).

### Measurement of serum levels of cytokines

The serum levels of IL-6 and IFN- γ were determined using a MAGPIX Milliplex kit (Merck, Darmstadt, Germany), according to the manufacturer’s instructions. Briefly, 25 μL of serum samples, standards, and controls were added to a 96-well plate comprising an equal amount of assay buffer for serum samples or serum matrix for standards and controls. Magnetic beads coated with antibodies against the target cytokines were added to each well, and the plates were incubated on a plate shaker overnight at 4 °C. After washing with washing buffer in the kit, the samples were reacted with biotinylated detection antibodies for 1 h and then with streptavidin–phycoerythrin for 30 min. After washing and addition of loading buffer from the kit, the samples were analyzed by the MAGPIX system (Luminex, Austin, TX, USA). The results are presented in Supplemental Fig. S3.

### Statistical analysis

Statistical analyses were performed using Prism 7 (GraphPad Software, San Diego, CA, USA). Differences were identified using unpaired *t*-tests or two-way ANOVA and were considered significant when *p* < 0.05. Data are presented as mean ± standard error of the mean (SEM). All experiments were performed at least twice to confirm the reproducibility.

### Ethical statement

All mouse experiments were performed with approval from the Animal Care and Use Committee of Hokkaido University following the Fundamental Guidelines for Proper Conduct of Animal Experiment and Related Activities in Academic Research Institutions under the jurisdiction of the Ministry of Education, Culture, Sports, Science and Technology in Japan. Body weight losses were monitored daily after infection, and mice were humanely euthanized when weight loss reached 25%.

## Supplementary information

Supplementary file1 (PDF 1761 kb)

Supplementary file2 (XLSX 66 kb)

## Data Availability

All data generated or analyzed during this study are included in this published article and its Supplementary Information Files.
