# Supplementary material for: Influenza virus infection affects insulin signaling, fatty acid-metabolizing enzyme expressions, and the tricarboxylic acid cycle in mice
Source: Sci Rep. 2020 Jul 2;10:10879. doi: 10.1038/s41598-020-67879-6 (PMC7331672; doi:10.1038/s41598-020-67879-6)

**Title**

Influenza virus infection affects insulin signaling, expression levels of fatty acid metabolizing enzymes, and the tricarboxylic acid cycle in mice

**Authors**

Marumi Ohno[a], Toshiki Sekiya[b], Naoki Nomura[a], Takuji Daito[a], Masashi Shingai[a], Hiroshi Kida[a*]

**Author's affiliation**

[a]Research Center for Zoonosis Control, Hokkaido University, Sapporo, Japan

[b]Global Station for Zoonosis Control, Global Institution for Collaborative Research and Education (GI-CoRE) Hokkaido University, Sapporo, Japan.

***Corresponding author**

Hiroshi Kida

Research Center for Zoonosis Control, Hokkaido University, Kita 20 Nishi 10, Kita-ku, Sapporo 001-0020, Japan

Tel.: +81-11-706-9500; Fax: +81-11-706-9500.

E-mail: kida@vetmed.hokudai.ac.jp

**Supplemental Figure S1. PCA plot of serum metabolites (control *vs.* PR8 at 6 dpi)**

Mice were intranasally inoculated with PBS alone or PBS comprising PR8 virus, and serum samples were collected at 1, 3, and 6 dpi. Relative levels of serum metabolites were evaluated by metabolome analysis. PCA plot of the metabolome data that characterizes the trends exhibited by the serum metabolite profiles of control (red) and PR8 virus-infected mice (PR8, green) at 6 dpi. Dots represent samples and circles represent the treatment groups (red for control, green for PR8).

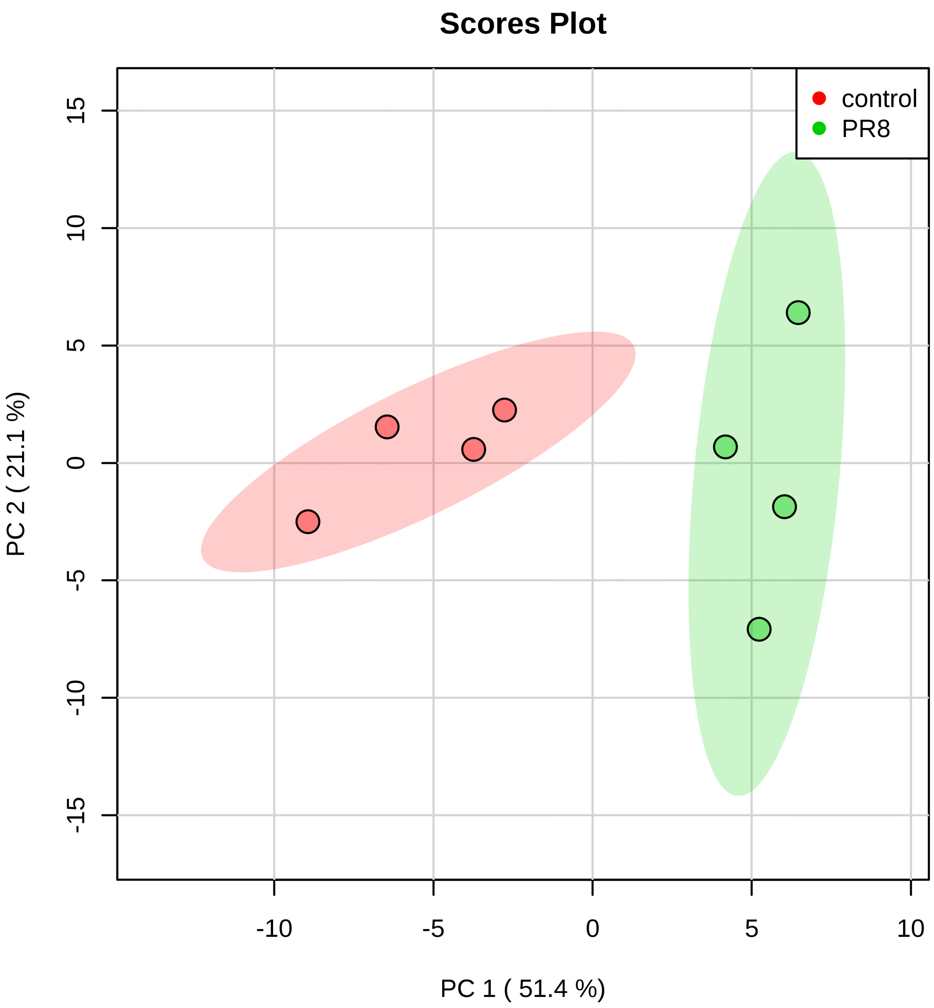

**Supplemental Figure S2. Effects of PR8 virus infection on insulin-induced Akt phosphorylation at different time points**

Mice were intranasally inoculated with PBS alone or PBS comprising PR8 virus. At 1, 3, and 6 dpi, mice were intraperitoneally injected with PBS or insulin after overnight fasting and liver samples were collected after 15 min for whole lysate preparation. Western blotting was performed to quantitate phosphorylated and total Akt protein levels in lysates. A representative Western blot analysis shows total Akt and Akt phosphorylated at Ser473 on the same membrane that was sequentially immunoblotted with corresponding antibodies. Relative Akt phosphorylation levels were calculated from band densities and are expressed relative to that from PBS-treated control mice. Average fold changes of the ratio of phosphorylated Akt to total Akt in control and PR8 virus-infected mice after insulin injection were 5.3 and 4.8 at 1 dpi, 5.0 and 1.9 at 3 dpi, and 5.6 and 2.4 at 6 dpi, respectively.

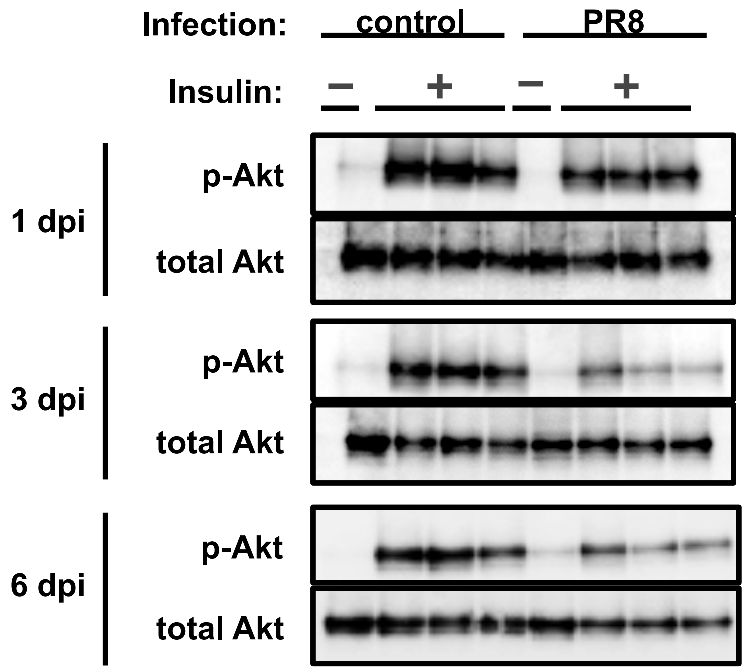

**Supplemental Figure S3. Serum cytokine levels (control vs PR8 at 3 and 6 dpi)**

Mice were intranasally inoculated with PBS comprising PR8 virus, and serum samples were collected at 3 and 6 dpi. The control mice received only PBS and their serum samples were collected at 6 day post injection. Serum levels of IL-6 and IFN-γ were measured using a MAGPIX Milliplex kit according to the manufacturer's instruction. Bars represent mean ± SEM of 4 animals; **p* < 0.05, one-way ANOVA, control vs. PR8 virus-infected mice.

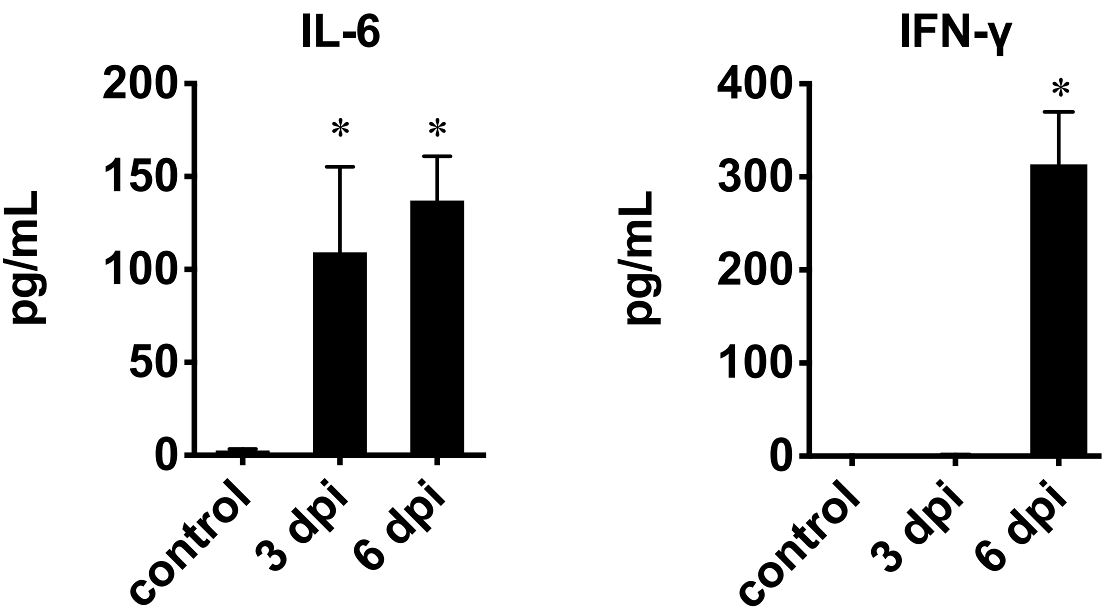

**Supplemental Figure S4. Original uncropped images of Western blots**

Full-length images of Western blots detecting p-Akt and total Akt. Boxes with broken lines indicate cropped images presented in Figure 4a (a, b) and Supplemental Figure S2 1 dpi (c, d), 3 dpi (e, f), and 6 dpi (g, h).

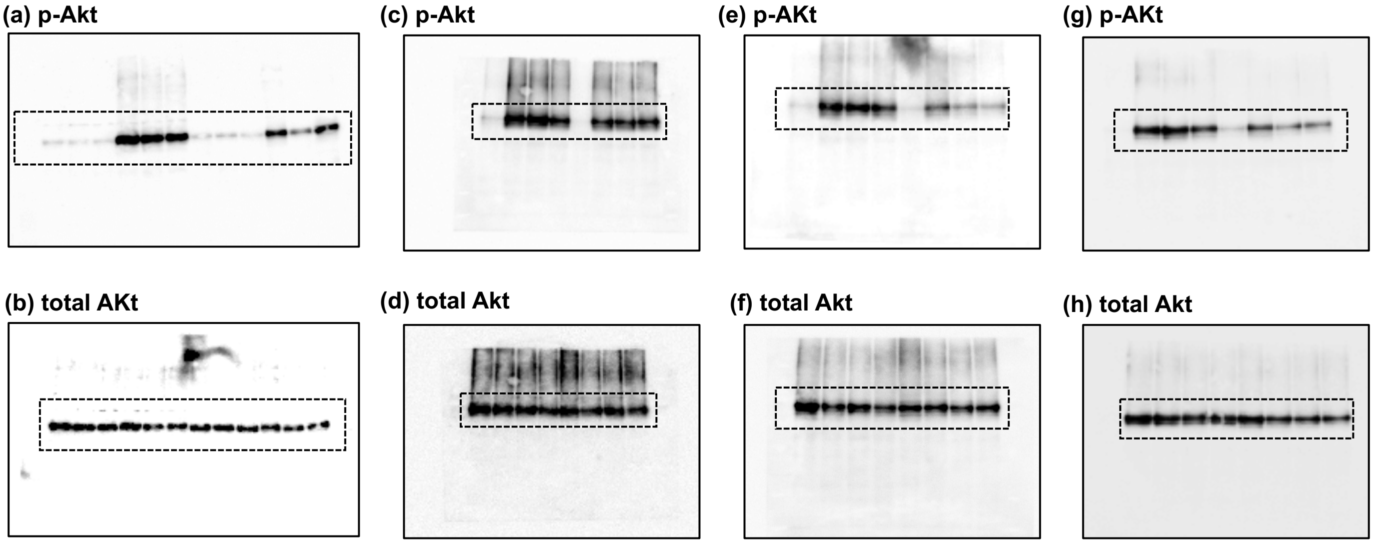

Supplement: Supplementary file 1 — Supplementary file1 (PDF 1761 kb) [file 41598_2020_67879_MOESM1_ESM.pdf]
